# Natural Compounds as Inhibitors of Aβ Peptide Aggregation: Chemical Requirements and Molecular Mechanisms

**DOI:** 10.3389/fnins.2020.619667

**Published:** 2020-12-22

**Authors:** Katiuscia Pagano, Simona Tomaselli, Henriette Molinari, Laura Ragona

**Affiliations:** NMR Laboratory, Istituto di Scienze e Tecnologie Chimiche (SCITEC), Consiglio Nazionale delle Ricerche – CNR, Milan, Italy

**Keywords:** NMR, amyloid-β protein, protein ligand interactions, self-association, natural compound, Alzheimer

## Abstract

Alzheimer’s disease (AD) is one of the most common neurodegenerative disorders, with no cure and preventive therapy. Misfolding and extracellular aggregation of Amyloid-β (Aβ) peptides are recognized as the main cause of AD progression, leading to the formation of toxic Aβ oligomers and to the deposition of β-amyloid plaques in the brain, representing the hallmarks of AD. Given the urgent need to provide alternative therapies, natural products serve as vital resources for novel drugs. In recent years, several natural compounds with different chemical structures, such as polyphenols, alkaloids, terpenes, flavonoids, tannins, saponins and vitamins from plants have received attention for their role against the neurodegenerative pathological processes. However, only for a small subset of them experimental evidences are provided on their mechanism of action. This review focuses on those natural compounds shown to interfere with Aβ aggregation by direct interaction with Aβ peptide and whose inhibitory mechanism has been investigated by means of biophysical and structural biology experimental approaches. In few cases, the combination of approaches offering a macroscopic characterization of the oligomers, such as TEM, AFM, fluorescence, together with high-resolution methods could shed light on the complex mechanism of inhibition. In particular, solution NMR spectroscopy, through peptide-based and ligand-based observation, was successfully employed to investigate the interactions of the natural compounds with both soluble NMR-visible (monomer and low molecular weight oligomers) and NMR-invisible (high molecular weight oligomers and protofibrils) species. The molecular determinants of the interaction of promising natural compounds are here compared to infer the chemical requirements of the inhibitors and the common mechanisms of inhibition. Most of the data converge to indicate that the Aβ regions relevant to perturb the aggregation cascade and regulate the toxicity of the stabilized oligomers, are the N-term and β1 region. The ability of the natural aggregation inhibitors to cross the brain blood barrier, together with the tactics to improve their low bioavailability are discussed. The analysis of the data ensemble can provide a rationale for the selection of natural compounds as molecular scaffolds for the design of new therapeutic strategies against the progression of early and late stages of AD.

## Introduction

Alzheimer disease (AD) is the main cause of neurodegenerative dementia (Selkoe, 2002b; Lopez and Kuller, 2019). Since the first descriptions of pre-senile dementia by Alois Alzheimer in 1907 (Alzheimer, 1907), the formation of extracellular senile plaques and intraneuronal fibrillary tangles have been regarded as the hallmarks of the neuropathology, as the behavioral symptoms of AD correlate with their accumulation (Selkoe, 1991; Armstrong, 2009; Bloom, 2014). The soluble species of these structures are amyloid-β (Aβ) peptides for plaques and tau protein for tangles. Aβ peptides are proteolytic fragments of the transmembrane amyloid precursor protein, whereas tau is a brain-specific, axon-enriched microtubule-associated protein (Black et al., 1996; Haass et al., 2012). The mechanism of Alzheimer onset and progression is complex and still not fully understood. Recent discoveries have revealed pathways that connect Aβ to tau in seminal steps of AD pathogenesis (Gotz et al., 2001; Lewis et al., 2001; Hurtado et al., 2010; Zempel et al., 2013). Aβ is upstream of tau in AD pathogenesis and triggers the conversion of tau from a normal to a toxic state, but there is also evidence that toxic tau enhances Aβ toxicity *via* a feedback loop (Leroy et al., 2012). Yet, substantial genetic evidences identify the misfolding and the extracellular aggregation of Aβ, mostly Aβ1–40 (Aβ40) and Aβ1–42 (Aβ42), as the main cause of AD progression (Hardy and Higgins, 1992; Hardy and Selkoe, 2002; Benilova et al., 2012; Selkoe and Hardy, 2016). The inhibition of Aβ self-assembly is therefore a promising therapeutic approach for the treatment of AD (Estrada and Soto, 2007).

**FIGURE 1:**
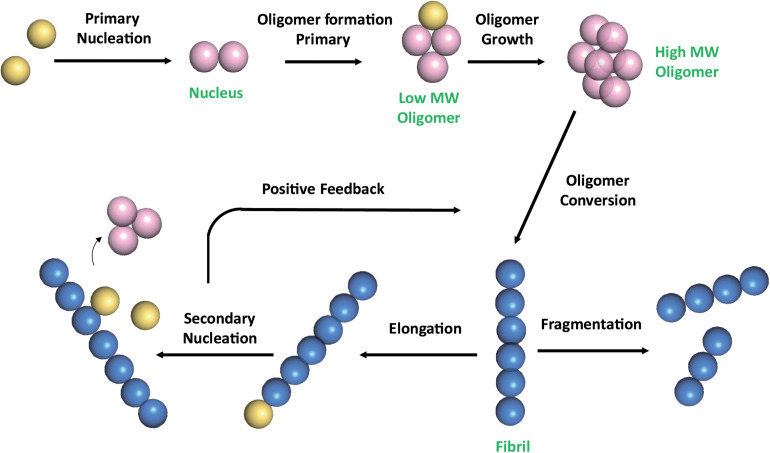
Schematic representation of Aβ self-association cascade. Aβ monomers initially combine to form a nucleus through primary nucleation process. Nuclei are defined as aggregates for which monomer addition is faster than its dissociation (Arosio et al., 2015). Addition of monomers to the nucleus, through the elongation process, results in the formation of oligomers, that are transient soluble intermediates that further elongate into fibrils. Fibrils can be disrupted through monomer-independent processes, such as fragmentation, with a rate depending only upon the concentration of existing fibrils. Fibril elongation by monomer addition and secondary nucleation depends on both the concentration of monomers and that of the existing fibrils (Linse, 2017; Scheidt et al., 2019). Once a critical concentration of mature fibrils has formed, the surfaces of existing fibrils catalyze the nucleation of new aggregates from the monomeric state (secondary nucleation). Secondary nucleation reaction overtakes primary nucleation as the major source of new diffusible oligomers (positive feedback) (Cohen et al., 2013). Color code: monomers are colored in yellow; nuclei and soluble transient oligomers are colored in pink; fibrils are colored in blue. The number of circles are for illustration purposes only and do not represent the actual number of subunits in the different species.

The multistep mechanism of Aβ monomers association is depicted in Figure 1.

Monomeric Aβ does not possess cellular toxicity under physiologically relevant concentrations, while soluble oligomers, which show very high heterogeneity in terms of size and structure, have been shown to exhibit substantial neurotoxicity (Knowles et al., 2014; Cremades and Dobson, 2018).

In the past, several compounds have been developed to reduce or prevent Aβ oligomerization and to destabilize disease relevant Aβ aggregates, however most of these molecules have shown serious side effects and poor permeability through the blood-brain barrier (BBB), the highly specialized endothelial cell membrane lining cerebral microvessels, which regulates the entry of plasma components into the central nervous system and ensures the export of potentially neurotoxic molecules from the brain to the blood (Abbott et al., 2010; Zenaro et al., 2017). Natural products, with their unique structural diversity, have come to focus as important sources of bioactive chemical domains, with minimal side effects and increased BBB permeability (Bui and Nguyen, 2017). A huge number of natural compounds have shown interesting beneficial effects on the onset and progression of different neurodegenerative diseases. Some classes, as tetracyclines and polyphenols, have the capability to interfere with the aggregation of several unrelated amyloidogenic proteins, such as α-synuclein (associated to Parkinson’s disease), islet amyloid polypeptide (IAPP, associated to type-2 diabetes), and it is likely they share partially overlapping mechanisms of action (Andrich and Bieschke, 2015; Giorgetti et al., 2018; Martinez et al., 2020). Several *in vitro* and *in vivo* studies have proven the therapeutic potential of a wide range of natural compounds against AD progression, however only a small subset of them have been shown to be efficient in preclinical and clinical studies (Andrade et al., 2019). A further critical issue remains the scarcity of reported experimental evidences on their mechanism of action. Some natural compounds have been proposed to attenuate the accumulation of Aβ peptide either by affecting several signaling cascades *via* the modulation of oxidative stress or by direct interaction with Aβ peptide and its self-assembled species (Sadhukhan et al., 2018).

In this review, we focus on the natural compounds able to bind monomeric, oligomeric or fibrillar Aβ species, as deduced from biophysical and/or structural biology approaches. The identification of the key chemical features of the inhibitors and the inhibiting mechanisms are reviewed to highlight the critical features to be addressed in targeting the toxic oligomeric species. The issue of natural compounds low bioavailability is examined and the most important tactics to overcome these limitations and allow successful conversion into therapeutics are discussed.

### Natural Compounds With Inhibitory Effects on Aβ Peptide Aggregation Pathways

A rich and important literature (Ma et al., 2020; Muscat et al., 2020b; Stefanescu et al., 2020) reports both experimental and computational studies, focused on the effects of natural compounds on different self-aggregated Aβ species. We have reviewed here those studies in which the proposed molecular mechanism of action is supported by a combination of biophysical approaches and experimental structural data. Most of the available data are based on optical spectroscopies, including circular dichroism (CD) and multiple types of fluorescence measurements, useful to estimate the progression of fibrillization, together with the beta content and hydrophobicity of the Aβ assemblies. In particular the use of dyes, such as thioflavin T (ThT), whose fluorescence intensity increases upon binding to the fibrils, is a popular method to detect amyloid fibrils formation. Dynamic light scattering (DLS), size exclusion chromatography (SEC), Transmission Electron Microscopy (TEM), Atomic Force Microscopy (AFM), provide important information on the size and the morphology of the aggregated species stabilized in the presence of the natural compounds. Conformation-specific antibodies, that recognize the different structural features of amyloidogenic oligomers and fibrils (Kayed et al., 2007; Perchiacca et al., 2012), are very informative to monitor/assess oligomer remodeling induced by natural compounds. High-resolution structural information on amyloid fibrils has only become available in recent years through progress in solid state NMR (ssNMR), cryo–electron microscopy (cryo-EM), and X-ray microcrystallography techniques (Chiti and Dobson, 2017). In particular cryo-EM approaches achieved atomic resolution structures of amyloid fibrils (Antequera et al., 2020; Ciudad et al., 2020). The resolution power of solution NMR spectroscopy contributed significantly to the definition of the molecular mechanism of action of aggregation inhibitors from natural sources. Indeed, NMR can highlight both Aβ residues involved in the interactions, as well as small molecule chemical groups stabilizing the binding. Thus, peptide-based and ligand-based NMR observation can be fruitfully employed to investigate the interactions of the natural compounds with NMR-visible or NMR-invisible Aβ species, respectively. Among ligand-based NMR approaches, saturation transfer difference (STD) experiment is a well-established homonuclear NMR technique that permits detection of transient binding of small molecule ligands to macromolecular receptors, such as Aβ oligomers, and to identify binding epitopes on the small ligand (Biet and Peters, 2001; Meyer and Peters, 2003). Among peptide-based NMR approaches, a very simple and widely employed experiment is ^1^H-^15^N Heteronuclear Single Quantum Coherence (^1^H-^15^N HSQC). It detects ^1^H signals that are directly bound to the ^15^N atoms, thus providing a fingerprint of the amide-NH backbone atoms, when a ^15^N labeled Aβ sample is available. This experiment is particularly suitable to investigate interactions, as it allows to map peptide residues involved in the binding sites and/or to identify conformational rearrangements, through the measure of changes in chemical shifts, linewidths and/or resonance intensities, upon ligand addition. The dark-state exchange saturation transfer experiment (DEST), has been developed to characterize, at atomic resolution, the interaction between an NMR-visible free peptide species, transiently bound to an NMR-invisible very high-molecular-weight macromolecular entity (Fawzi et al., 2011). Specifically, DEST NMR methods allowed to expand the knowledge of the determinants of the interaction between Aβ monomers with the NMR-invisible Aβ assemblies and with models of cellular membranes (Ahmed et al., 2019). Finally, simple ^1^H NMR approaches, exploiting the time-dependent decrease of NMR peak intensities, can be employed to derive the Aβ aggregation kinetics (Liu et al., 2012; Pagano et al., 2019). Docking and MD studies have been reported for the majority of the compounds, however a comparative analysis of the results is difficult, as simulations were run starting from different monomeric, oligomeric, fibrillar structural models.

The review summarizes results on natural compounds tested on both Aβ40 and Aβ42 isoforms, as they represent the two major components of the amyloid deposits in the brain (Haass and Selkoe, 1993). The longer Aβ42 is considered the most pathogenic, in view of its greater tendency to aggregate and form toxic oligomers (Hou et al., 2004; Qiu et al., 2015). However, Aβ40 is more abundant than Aβ42 (9:1) in the biological fluids (Qiu et al., 2015). In addition, Aβ40 was found in the amyloid deposits of patients affected by cerebral amyloid pathology, which is considered an early step in AD pathogenesis (Garcia-Vinuales et al., 2020; Greenberg et al., 2020), suggesting that both isoforms must be taken in consideration in search of neuroprotective agents.

For each natural compound with Aβ aggregation inhibitory properties, the main biophysical data available in the literature are reported in the following paragraphs and summarized in Table 1.

**TABLE 1:** Natural compounds with antiaggregation effects on Aβ peptide: chemical structure and summary of the methods employed to describe the inhibitory action.

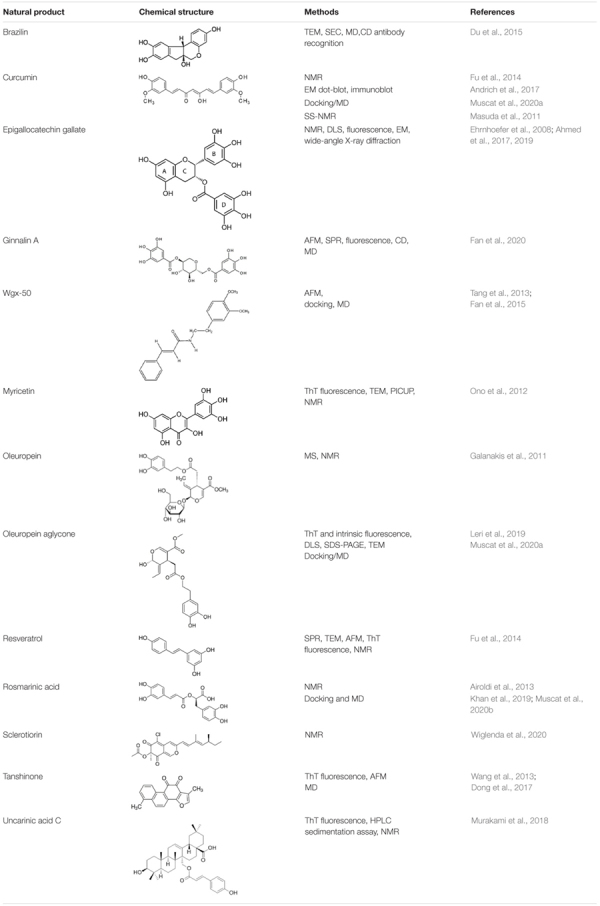

***Brazilin***, is a natural compound extracted from *Caesalpinia sappan*.

Biochemical, biophysical, cell biological and molecular simulation methods were applied to investigate Brazilin inhibitory effects on Aβ42 fibrillogenesis and cytotoxicity (Du et al., 2015). TEM data indicated that Brazilin inhibited fibril formation in favor of granular aggregates at Brazilin: Aβ ratio of 10. Brazilin induced aggregates were investigated by SEC revealing two elution peaks, one corresponding to large soluble aggregates with MW above 75KDa and one corresponding to monomeric Aβ42. CD spectra of Aβ42 detected the presence of β-sheet structures either in the presence and absence of brazilin, however, assays in the presence of antibodies demonstrated that Aβ42 aggregates modulated by brazilin were structurally distinct from the toxic oligomers. Time-dependent dot blot assays, carried out using antibodies A11 (specific for toxic on-pathway Aβ oligomers), and 6E10 (recognizing Aβ independently of its conformations), showed that, in the presence of brazilin, A11-immunoreactive oligomers were efficiently suppressed, while 6E10 antibody detected both brazilin-treated and untreated Aβ42.

Lactate dehydrogenase (LDH) cytotoxicity assays were further carried out using SH-SY5Y cell line, a human derived neuroblastoma cell line presenting many of the biochemical and functional features of human neurons, widely used as a human neuronal cell model in the cytotoxicity studies of AD. Co-incubation of Aβ42 monomers and brazilin decreased the Aβ42-induced cell death in a dose-dependent manner, as evidenced by the decrease of LDH release. Molecular simulations, employing Aβ 17–42 pentamer as starting state, indicated that brazilin could inhibit Aβ42 fibrillogenesis by directly binding to Aβ42 species *via* hydrophobic interactions and hydrogen bonding, and further remodeled mature fibrils by disrupting the intermolecular salt bridge D23-K28 *via* hydrogen bonding. Aβ residue mainly involved in interaction were L17, F19, F20, and K28 (Du et al., 2015).

***Curcumin (Cur)***, is a polyphenol extracted from rhizome of the Turmeric/Curcuma longa plant.

Curcumin was reported to dose-dependently inhibit the formation of Aβ fibrils from Aβ40 and Aβ42 and their extensions, as well as to destabilize preformed fibrils (Hamaguchi et al., 2010). At variance, other authors suggested, on the basis of Western blot, TEM and fluorescence data, that curcumin promotes fibrillization by shifting Aβ42 fibrillation pathway away from prefibrillar aggregates (Necula et al., 2007; Caesar et al., 2012). Masuda et al. (2011) investigated the binding of curcumin to fibril structures by ssNMR employing selectively ^13^C labeled Aβ42 residues at the N-terminal and in the 17–21 region. 2D ^13^C–^13^C correlation experiments indicated that residues V12 and L17–A21 were mainly involved in the interaction with curcumin. The relevance of the paper is related to the demonstration that the polyphenol interacts with Aβ42 fibrils. Dot blot assays with the A11 antibody showed that Cur inhibited the formation of high molecular weight Aβ oligomers (Necula et al., 2007). Along the same line, Fu et al. (2014) demonstrated by AFM that when Cur was co-incubated at a 1:1 molar ratio with Aβ peptide, the oligomers height were capped at ∼2.5 nm, thus indicating that Cur stabilizes the low molecular weight oligomers (with heights measured by AFM of ∼1.0-2.5 nm) rather than high molecular oligomers (height 3–5 nm) additionally observed in its absence.

Curcumin was also shown to bind monomeric species and/or low MW oligomers. 2D ^1^H-^15^N HSQC NMR studies indicated that Aβ42 residues involved in the interaction are A5, S8, G9, K16, K17, D23, and I31 (Ono JBC 2012). Similar regions (E3, F4, R5, Q15, K16, L17, F20) were mapped by Fu et al. (2014).

***Epigallocatechin gallate (EGCG*)**, is extracted from green tea.

Solution NMR studies indicate that EGCG is able to bind weakly to Aβ monomers while it displays higher affinity toward oligomers (Kd is one order of magnitude lower for oligomers with respect to monomers). ^1^H-^15^N HSQC spectra showed a dose-dependent change in Aβ40 chemical shifts upon EGCG addition, suggesting that EGCG interacts with monomers. The most pronounced chemical shift changes were observed for residues within the two β-strands seen in the Aβ40 protofibrils. However, the chemical shift projection analyses pointed out that EGCG binding to monomer is most likely nonspecific (Ahmed et al., 2017).

DLS changes observed upon EGCG addition to Aβ40 peptide are consistent with the ability of EGCG to stabilize Aβ40 oligomers starting from either monomers or soluble high molecular weight protofibrils (Bieschke et al., 2010; Ahmed et al., 2017). Electron microscopy (EM) images indicated that, after EGCG addition, the protofibrils are remodeled into smaller spherical oligomers. DLS and EM evidenced the EGCG-induced morphological remodeling, while a combination of several cutting-edge NMR approaches were employed to investigate the same remodeling mechanism at a per-residue level. Specifically, EGCG induced a net inhibition of direct Aβ40 monomer–protofibril contacts in the β1 strand, while a net induction of more engaged direct Aβ40 monomer-protofibrils contacts was observed for the N-terminal segment. The dual effect displayed by EGCG explains the remodeling of mature amyloid assemblies into smaller, non-toxic aggregates without the release of transient Aβ monomers. As a result EGCG stabilizes seeding-incompetent, off-pathway oligomers (Ahmed et al., 2017 and references therein; Ehrnhoefer et al., 2008; Ahmed et al., 2019), interfering with secondary nucleation events known to generate toxic Aβ assemblies (Ahmed et al., 2017).

The comparative analysis of Aβ soluble assemblies (Aβn) prepared in the absence or presence of a set of catechins including EGCG, epigallocatechin (EGC) and epicatechin (EC), allowed to correlate cellular toxicity with the unique molecular signatures of Aβn induced by the different natural compounds (Ahmed et al., 2019), indicating that Aβn toxicity is regulated by the solvent exposure of hydrophobic surfaces. The interactions of selected Aβ assemblies with membrane biomimetics (SUVs composed of a mixture of DOPE: DOPS: DOPC lipids) were further characterized through TEM and NMR approaches proposing a model for oligomer toxicity (Ahmed et al., 2019) (see later, section “Identification of Aβ Regions Affected by Natural Compounds and Oligomer Remodeling Mechanisms”).

***Ginnalin A (GA)***, is isolated from the red maple. GA addition to Aβ42 lowers the ThT fluorescence intensity at the plateau and lengthens the lag phase of the aggregation in a dose-dependent manner. Surface plasmon resonance (SPR) experiments demonstrate that GA binds to Aβ42 monomers and/or pentamers (Fan et al., 2020). AFM data confirmed that co-incubation of Aβ42 with GA drastically altered the Aβ42 aggregation kinetics and protofibrils were absent even after 6 h co-incubation, while irregular amorphous aggregates, instead of mature fibrils, were observed after 24 h. The capability of GA to disintegrate preformed fibrils was further tested by AFM, showing that preformed fibrils could be efficiently disaggregated into many small and amorphous aggregates depending on GA/Aβ42 ratio, with the size of the amorphous aggregates decreasing with the increase of GA concentration. Time-lapse CD, performed without and with GA addition to Aβ42, indicated that the random coil/α-helix to β-sheet conformational conversion, a crucial step in fibrillogenesis, was inhibited in a concentration-dependent manner. Further data on the interaction GA/Aβ42 at a per-residue level were based on MD simulations suggesting that GA would target monomers residues 17–21, 35, 38, reported to be essential for β-sheet formation and stabilization (Hoyer et al., 2008; Ahmed et al., 2010). The authors propose that GA can target Aβ42 fibrillogenesis *via* different mechanisms, namely by binding to monomers at the early nucleation phase, preventing Aβ–Aβ associations, and at the later growth phase. Pre-incubation of Aβ42 solution with GA give rise to oligomers that do not interact or do not perturb the integrity of cellular membranes, as deduced from toxicity tests on SH-SY5Y cells.

***Wgx50***, is a natural compound extracted from Sichuan pepper (*Zanthoxylum bungeanum*) that is able to inhibit Aβ-induced neuronal apoptosis, reduce neuronal calcium toxicity, decrease the accumulation of Aβ oligomers in the cerebral cortex, and improve the cognitive abilities of mice (Tang et al., 2013, 2014; Guo et al., 2014; Shi et al., 2016). AFM investigation showed that wgx-50 can directly inhibit Aβ oligomers. Tang et al. (2013) demonstrated that when Aβ42 peptide was co-incubated with wgx-50, no high density plaques and fibrils were observed, oligomers disassembled and only a few fibrils remained, suggesting that wgx-50 could prevent Aβ fibrils growth.

The binding of wgx-50 on Aβ fibrils was investigated by docking and MD simulations. The small molecule was docked on Ab17–42 pentameric U-shaped fibril (Fan et al., 2015), and the analysis of the following MD simulations highlighted three possible stable binding sites. Two sites interested the two hydrophobic grooves on the surface of Aβ protofibril at the level of residues F20 and V18 for the first one, and residues I31 and M35 for the second one. The binding on these two sites made no significant changes in Aβ structures, but they corresponded to regions responsible for the intermolecular protein-protein assembly of amyloid (Nasica-Labouze et al., 2015). In the third binding site, wgx-50 was packed against the side chains of I32 and L34 in the interior of the protofibril, and caused destabilization of the structure, disrupted the D23-K28 salt bridges, and partially opened the tightly compacted two β-sheets.

***Myricetin (Myr)***, is a flavonol found in many foods of vegetal origin, including tea, onions, cocoa and red wine. Myr was shown to prevent the growth of Aβ40 and Aβ42 amyloid aggregates *in vitro* and to destabilize preformed fibrils, as demonstrated by ThT fluorescence and TEM analysis (Ono et al., 2003, 2004, 2005). Myr can block Aβ40 and Aβ42 oligomerization, as shown by photochemical cross-linking method (PICUP) (Ono et al., 2012). NMR studies demonstrated that Myr is able to bind Aβ42 monomers, promoting significant chemical shift perturbations of ^1^H-^15^N HSQC spectra at the level of R5, V12, H13, K16-D23, I31, and I32. Interestingly, the authors observed the selective broadening of few amide peaks (R5, V12, K16, and V18), suggesting a stronger binding in this region. MTT, LTP, and LTD assays established that the phenol remodeled Aβ oligomer induced lower cellular and synaptic toxicities (Ono et al., 2012).

***Oleuropein (OE) and Oleuropein aglicone (OleA)***. OE is a polyphenol extracted from the fruits and leaves of *Olea europaea L.* endowed with a wide range of beneficial pharmacological effects, such as antioxidant, anti-inflammatory, antiatherogenic, antibacterial, and anticancer properties (Bazoti et al., 2008 and references therein). Mass spectrometry (MS) experiments showed that OE interacts non-covalently with Aβ, targeting the Aβ amino acid segment F4–K28 (Bazoti et al., 2008). NMR titration experiments demonstrated the ability of OE to interact with Aβ40 monomer and/or low MW oligomers, with the region H14-F20 identified as the binding epitopes (Galanakis et al., 2011), in agreement with MS results.

Oleuropein aglicone (OleA) is an OE derivative, devoid of the glucose moiety. OleA is the most abundant and typical phenolic components of extra virgin olive oil. Recent data indicated that OleA interferes *in vitro* with the aggregation pathway of amyloidogenic peptides and proteins, including amylin, tau protein, transthyretin (Leri et al., 2016), β2-microglobulin, α-synuclein, and Aβ42 peptide (Leri et al., 2019 and references therein). ThT, DLS, SDS-PAGE, TEM analysis suggested that OleA favors the growth of minute protofibrils, inhibiting their further development along the fibrillation pathway (Daccache et al., 2011; Leri et al., 2019). Intrinsic fluorescence quenching experiments of Aβ42 Tyr10, in the presence of acrylamide, demonstrated that OleA binds and stabilizes solvent-exposed oligomeric intermediates, delaying fibril formation. Amyloid assemblies grown in the presence of OleA were unable to bind the cell membrane at the GM1 level, as indicated by FRET efficiency, thus exhibiting low cytotoxicity (Leri et al., 2019). Altogether the data highlight the ability of OleA to induce specific and essential changes in the monomeric peptide or oligomeric assemblies to redirect the aggregation process toward off-pathway and harmless final products.

Docking of OleA to the S-shaped fibril (Fandrich et al., 2018), followed by 150 ns MD simulations, suggested that OleA localizes between adjacent receptor chains, mostly interacting with V18–V24 and N27–I31. The type of established interaction reduces the percentual content of beta sheets, the order parameter value and the inter-chain interaction area if compared to the wild type structure, thus inducing a considerable destabilizing effect on the whole amyloid fibril (Muscat et al., 2020a).

***Resveratrol (RES)*** is a natural non-flavonoid polyphenolic molecule found in many fruits, vegetables, tea, and wine (Berman et al., 2017). RES, thanks to its numerous therapeutic effects, has been studied as therapeutic agent for cardiovascular disease, obesity, diabetes, and for neurological disorders, including Alzheimer’s diseases (Tellone et al., 2015; Martin, 2017; Rauf et al., 2018). SPR analysis demonstrated that RES can directly bind to both monomeric and fibrillar Aβ40 and Aβ42 peptides (Ge et al., 2012). Solution NMR data confirmed the affinity of RES for monomeric species and/or small oligomers.

RES can dose-dependently inhibit Aβ42 fibril formation, as demonstrated by TEM. The co-incubation of Aβ42 with RES leads to the formation of oligomers, suggesting that RES does not inhibit Aβ42 oligomer formation (Feng et al., 2009). Fu et al. (2014) demonstrated by single touch AFM that, when the peptide was co-incubated with RES, low MW oligomers (20 kDa, tetramer) were stabilized. The remodeled RES-capped oligomers showed a reduced cell toxicity, as deduced by MTT assay (Feng et al., 2009; Fu et al., 2014).

The analysis of ^1^H-^15^N HSQC experiments, showed that resveratrol binds to the peptide N-terminus, as discussed in details in section “Identification of Aβ Regions Affected by Natural Compounds and Oligomer Remodeling Mechanisms.”

***Rosmarinic acid (RA)*** is a phenolic compound present in several plants of the *Lamiaceae* family.

ThT fluorescence and EM studies indicated that RA dose-dependently inhibited fibril formation of Aβ40 and Aβ42 and destabilized pre-formed fibril (Ono et al., 2004). TEM images indicated the ability of RA in inhibiting Aβ42 fibril formation (Sun et al., 2019).

Solution NMR studies highlighted the ability of RA to bind Aβ42 oligomers (Airoldi et al., 2013). STD and trNOESY NMR experiments indicated that RA aromatic groups have the highest involvement in the interaction with the oligomers.

Further data on the interaction Aβ40/RA were based on docking studies. The models obtained using Autodock starting from the ssNMR structure of Aβ40 [PDB ID: 2m4j (Lu et al., 2013)] showed that the complex is stabilized by seven hydrogen bonds involving OH group of dihydroxyphenyl with H13 and L17 and OH group of 2-propenyl with G37. RA interactions thus mask hydrophobic residues of the peptide, thus preventing aggregation (Khan et al., 2019).

Docking of RA to the S-shaped fibril (Fandrich et al., 2018), followed by 150 ns MD simulations suggested that RA mostly interacts with the chain edge, establishing interactions with residues E11-H14 and I32-L34, without inducing remarkable protein conformational changes (Muscat et al., 2020a).

***Tanshinones*** are lipophilic compounds extracted from the roots of *Salvia miltiorrhiza Bunge*. Tanshinone I (TS1) and tanshinone IIA (TS2) are the two most abundant components in the SMB herb. ThT and AFM data clearly demonstrated that they both inhibited amyloid formation at the early lag phase and at the later growth phase (Wang et al., 2013), with TS1 showing better inhibitory potency than TS2. Cell-toxicity experiments reported on a significant level of tanshione-induced protection of cultured SH-SY5Y cells, indicating that tanshinones are effective inhibitors of Aβ-induced *in vitro* toxicity. Alternative modes of interactions have been proposed, based on simulations employing different fibril models as starting states (Wang et al., 2013; Dong et al., 2017). Most recent MD simulations in explicit solvent, using a combination of secondary structural analysis, MM-PBSA binding energy calculations, and radial distribution functions computations, indicated that the charged residues within the disordered N-terminus tail of Aβ40 and Aβ42 are the preferred targets of tanshinones (Dong et al., 2017). According to the simulations, TS molecules favor a disaggregation mechanism driven by the interaction between TS and the N-terminal region of Aβ42 fibril, with a shift of the β1 region and a consequent fibril twist around its fibril axis. As a result, the β-sheet content is reduced and the stability of Aβ42 fibril decreased, hampering the lateral association of Aβ aggregates, inhibiting fibril growth.

***Sclerotiorin (SCL)*** is a chlorine-containing azaphilone-type natural product, that was first isolated in 1940 from *Penicillium sclerotiorum* (Curtin and Reilly, 1940).

Several experimental assays (such us filter retardation assays, EM, CD, ANS) demonstrated unequivocally that SCL perturbs early events in the fibrillar assembly process, leading to the stabilization of small structures with low β-sheet content and a low propensity to form fibrillar aggregates (Wiglenda et al., 2020). Specifically, immunoblot assay and EM indicated that SCL stabilizes Aβ42 oligomers migrating at 70–500 KDa. NMR studies showed that SCL addition to Aβ peptide prevents the broadening, due to aggregation, of ^1^H 1D and ^1^H-^15^N HSQC spectral resonances, holding Aβ42 peptides in a soluble state. At variance, Aβ42 resonances of untreated samples broaden as a function of time, as a consequence of the formation of high MW NMR invisible species. In line with the reported results, residue level analysis through ^1^H-^15^N HSQC spectra did not show any chemical shift perturbation, indicating that SCL targets NMR invisible Aβ species, namely high molecular weight oligomers.

WaterLOGSY experiments assessed the interaction between SCL and Aβ42 peptides. In these NMR experiments, the bulk water magnetization is excited and transferred during the NOESY mixing time to the bound ligand *via* different mechanisms. In the WaterLOGSY experiment positive effects were detected only at SCL:Aβ42 of 10 indicating that SCL weakly interacts with Aβ42 molecules in solution. Ion mobility-mass spectrometry (IM-MS) experiments were further employed to separate Aβ42 species based on differences in their overall size, as well as their charge. IM-MS further provides an absolute rotationally averaged collision-cross section (CCS), which allows the prediction of secondary structural changes within aggregating systems. The results indicated that SCL binding leads to conformational compaction at least in a small fraction of Aβ42 monomers, which influences their conversion rate into aggregation-competent β-sheet-rich fibrils. The authors propose two potential mechanisms of interaction based on direct association of the hydrophobic diene side chain of SCL with the central or the C-terminal hydrophobic regions of Aβ42 peptides, thus facilitating β-sheet formation and promoting the intermolecular association of monomers. Alternatively, the SCL oxygenated bicyclic ring system could form covalent bonds with polar amino acids in the peptide, such as K16 or K28 that are important for the formation of a stable, aggregation- competent b-hairpin structure. IM-MS data provide support to this second hypothesis. The data indicate that SCL binding leads to oligomers remodeling and stabilization of small structures with low β-sheet content, that are up-taken in mammalian cells to a lower extent and exhibit reduced toxicity.

***Uncarinic acids*** are triterpenoids derived from a Japanese medicinal herb *Uncaria rhynchophylla*. A combination of ThT fluorescence experiments and sedimentation assay using HPLC indicated that they inhibit the nucleation phase of Aβ42 aggregation (Murakami et al., 2018). Solution NMR studies suggested that Uncarinic acid C can bind Aβ42 monomeric species or low molecular-weight oligomers (dimers, trimers, etc.), making contacts with residues R5, H13-L17. Ion mobility-mass spectrometry evidenced the ability of triterpenoids to form a salt bridge between their carboxy group and K16 and K28. The results highlighted the relevant role of carboxy groups whose direct interaction with monomers, dimers and trimers suppressed further oligomerization (Murakami et al., 2018).

### Ligand-Epitopes Involved in the Interaction With Aβ

Small molecules from natural sources, effective in inhibiting Aβ aggregation, belong to different classes of chemical compounds, although polyphenols appear to be the most represented class (see Table 1). They can affect one or multiple aggregation stages by directly interacting with Aβ peptide through non-covalent and/or covalent interactions (Ma et al., 2020). Polyphenols ability to hamper Aβ aggregation has been attributed to the combined action of the phenolic moieties, that can π-π stack or interact hydrophobically with Aβ aromatic residues and insert into the space of Aβ aggregates, and/or interact *via* hydrogen bonding through phenolic hydroxyl groups (Fan et al., 2020 and references therein). We focus here on those reports dealing with NMR experiments dedicated to prove the binding epitopes of the natural compound.

Ligand-based NMR spectroscopy, especially STD approaches, were able to dissect the specific ligand binding epitopes for the interaction with amyloid peptides and to select the major components of a natural product interacting with peptide oligomers (Airoldi et al., 2013; Sironi et al., 2014).

In the case of EGCG, NMR approaches demonstrated that the flavan-3-ol unit of catechins is essential for interaction (Sironi et al., 2014) and the epitope mapping, derived from 1D STD, combined with transfer NOESY analyses, revealed that EGCG rings A and D (see Table 1) provide the primary contact sites with Aβ40 oligomers, while rings B and D undergo significant reorientation, with respect to the plane defined by A and C rings, upon interaction with Aβ40 assemblies (Ahmed et al., 2017).

A combination of NMR and synthetic approaches allowed dissecting the differential contribution to Aβ42 recognition and binding of the aromatic entities of rosmarinic acid (Airoldi et al., 2013) and chlorogenic acid extracted from coffee (Ciaramelli et al., 2018). NMR approaches have also been extensively employed to investigate the recognition mechanism of flavonoids, widely available in natural foods, for Aβ42 oligomers (Guzzi et al., 2017). Quercetin has been identified, on the basis of the STD NMR data, as the best binder, whereby its aromatic A ring represented the region mostly involved in the interaction.

Many polyphenols share a catechol moiety, which plays a role in Aβ interaction. Indeed, it was demonstrated that the inhibiting activity increased depending on the number of catechol moieties (Tsunoda et al., 2018). When the catechol belongs to a flavonoid, the inhibiting activity has been also related to the formation of Michael adducts with the side chains of K16 and K28 through flavonoid autoxidation (Murakami and Irie, 2019).

A combination of biophysical and docking method additionally identified a guaiacol moiety as an important requirement for the antiaggregating activity of natural based polyphenols (Tomaselli et al., 2019).

Structural planarity is a further feature shared by many inhibitors. Planar molecules are able to establish π–π stacking with Aβ aromatic residues, thus destabilizing the intermolecular region in Aβ42 aggregates (Murakami and Irie, 2019). Curcumin, tanshinone, and uncarinic acid C share this feature.

Uncarinic acid C belongs to another class of inhibitors, namely sterols. In addition to their interference through the planar hydrophobic structure, it has been proposed that the carboxylic acid group has an important role for the inhibitory activity, as it can establish a salt bridge with K16 and K28 side chains (Bittner, 2006; Murakami et al., 2018), thus hampering further elongation.

The variety of chemical structures observed across the discussed inhibitors underlines once more the importance of natural products as a rich source of bioactive chemical domains, relevant in the discovery and development of new drugs regulating amyloidogenesis.

### Identification of Aβ Regions Affected by Natural Compounds and Oligomer Remodeling Mechanisms

The comparative evaluation of the kind and toxicity of the Aβ oligomeric species stabilized in the presence of the small molecules, represents a further issue to be considered in ranking the behavior of natural compounds.

Natural compounds can affect amyloid aggregation pathways, targeting different steps of the amyloid aggregation cascade (Figure 1; Ahmed and Melacini, 2018; Wiglenda et al., 2020). The inhibitors action have been attributed to (a) the inhibition of monomers association leading to small aggregates; (b) remodeling of Aβ oligomers producing off-pathway seeding incompetent species; (c) inhibition of secondary nucleation (Ahmed and Melacini, 2018). Although many natural compounds have been reported to accomplish their action through multiple molecular mechanisms (Fan et al., 2020), the driving mechanism depends on the relative affinities of the inhibitors for Aβ species. For instance, in the case of EGCG, the mechanism of amyloid inhibition is driven by the preferential binding to Aβ oligomers (Ahmed et al., 2017; Ahmed and Melacini, 2018).

Soluble oligomers have been recognized as the most neurotoxic species (Klein et al., 2001; Knowles et al., 2014), thus making the targeting of these disease relevant species a valuable therapeutic solution. Many natural compounds can bind oligomers and stabilize off-pathway species. The therapeutical relevance of these interactions can be evaluated testing the citotoxicity of the resulting oligomers. Indeed, it is widely reported that amyloid aggregate cytotoxicity requires the primary interaction with the cell membrane (Campioni et al., 2010; Benilova et al., 2012) and the poor cytotoxicity is mainly a consequence of their inability to penetrate it (Kayed et al., 2004).

Aβ soluble oligomers, displaying a very high heterogeneity in size distribution, have been classified in two main groups: low and high molecular weight oligomers, named LMW and HMW, respectively (Fu et al., 2014). Aβ42 low MW oligomers (∼20 kDa), are essentially composed of tetramers, with smaller amounts of dimers and hexamers. High MW oligomers (∼54–60 kDa or higher) appear to be more toxic *in vitro* and *in vivo* compared to Aβ42 monomers, low MW oligomers, and fibrils (Lesne et al., 2006; Frydman-Marom et al., 2009). Natural compounds were shown to interact with both LMW and HMW oligomers. The residue level description of the interactions between natural compounds with LMW oligomers, that are NMR-visible species, can be based on the chemical shift mapping derived from ^1^H-^15^N HSQC spectra analysis. The NMR characterization of the interactions with soluble HMW oligomers (NMR invisible species) is more challenging and need dark state saturation transfer approaches.

A few natural compounds, namely resveratrol, curcumin, myricitin, Uncarinic Acid C, and oleuropein have been reported to bind monomers or low molecular weight oligomers. Specifically, Fu et al. (2014) demonstrated that resveratrol interacts with Aβ42 low molecular weight oligomers (∼ 20 kDa, tetramer). Residue level NMR analysis through ^1^H-^15^N HSQC experiments indicated that the largest chemical shift changes were observed for residues at the N-terminus and middle region of the Aβ42 sequence (E3, R5, S8, Y10, Q15, K16, L17, F20), while the hydrophobic C-terminal residues were substantially unperturbed. A similar pattern of perturbations (E3, F4, R5, Q15, K16, L17, F20) was observed in the presence of curcumin, which is also able to make direct interaction with Aβ peptide and stabilize Aβ species endowed with lower cytotoxicity (Fu et al., 2014). The binding of both molecules predominantly occurred at the positions of polar residues, belonging to N-terminal region, which are surface exposed and water accessible, as probed by ^1^H-^15^N HSQC-NOESY, which measures exchange between the backbone amide protons and water (Fu et al., 2014).

Myricitin can bind Aβ42 monomers and or/LMW oligomers as evidenced by marked chemical shift perturbations and amide resonances line broadening for R5, V12, K16, and V18 (Ono et al., 2012).

The sterol Uncarinic Acid C was shown to interact with the Aβ42 monomer or LMW oligomers (dimers, trimers, etc.) (Murakami et al., 2018) with R5 and the segment H13-L17 being the most perturbed.

NMR and MS studies showed that oleuropein interacts, non-covalently, in a 1:2 stoichiometry with Aβ40. Enzymatic cleavage of the Aβ:OE complex prior to ESI-MS analysis, indicated that OE interacts with Aβ regions F4–E11, V12–E22, and F17–K28 (Bazoti et al., 2008). NMR titration studies demonstrated that OE interacts with monomer or low MW oligomers, based on the observation of ^1^H and ^15^N chemical shift perturbation of selected residues (D7, H14, Q15, K16, V18, F19, F20, D23, S26, N27, K28) in ^1^H-^15^N HSQC spectra of the NMR-visible species (Galanakis et al., 2011).

**FIGURE 2:**
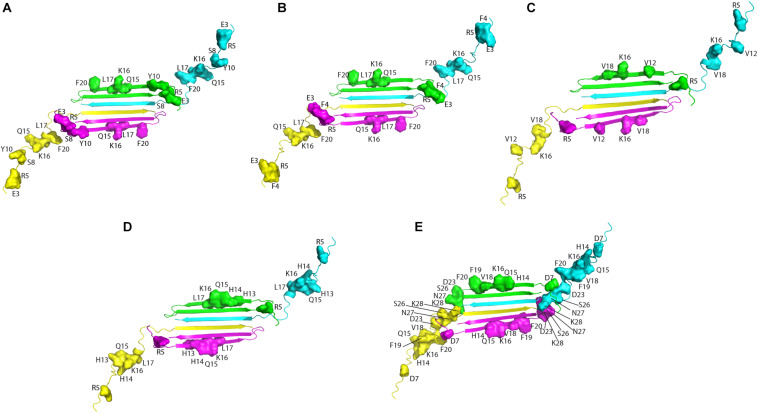
Aβ residues affected by the addition of the natural compounds, as deduced from ^1^H-^15^N HSQC experiments, are mapped on the structure of Aβ42 tetramer (PDB ID: 6RHY). The picture summarizes data reported for: resveratrol (Fu et al., 2014) **(A)**, curcumin (Fu et al., 2014) **(B)**, Myricetin **(C)** (Ono et al., 2012), uncarinic Acid C (Murakami et al., 2018) **(D)**, and Oleuropein (Galanakis et al., 2011) **(E)**. Four different colors are employed to distinguish the four Aβ subunits forming the tetramer. Chains **(A–D)** of the deposited PDB structure are colored in green, cyana, purple, and yellow, respectively. Residues perturbed by the addition of the natural compounds are highlighted as closed surfaces on the tetramer cartoon.

Altogether, solution NMR data on the interaction of natural compounds with low molecular weight oligomers highlight a common perturbed region located at the N-terminal and β1 strand. It is interesting to map the residues on the recently published solution NMR structure of the Aβ42 oligomer (tetramer) formed in a membrane-mimicking environment (Ciudad et al., 2020). This is the first atomic view of Aβ42 oligomers based on experimental data which is greatly helpful to decipher the mechanisms of pathogenesis and design therapeutics (Darling and Shorter, 2020). The tetramer comprises a β-sheet core made of six β-strands, connected by only two β-turns, leaving two short and two long, flexible N-termini. Notably, residues relevant for the interaction of the natural compounds, as deduced from NMR interaction studies, are all located in highly accessible regions, at the two long flexible N-termini and at the edge of the β-sheet core (Figure 2).

The authors propose, based on MD simulations that the N-termini of the Aβ42 tetramers and octamers, with all their charged residues, are required to traverse through the hydrophobic core of the bilayer, leading to the formation of lipid-stabilized pores. The relevance of the N-terminal tail for membrane interaction and the associated oligomer toxicity has been previously highlighted by many studies (Benilova et al., 2012; Murray et al., 2017; Ahmed et al., 2019). On the basis of the comparative analysis of data reported in the literature (Figure 2), it can be proposed that the binding of the small molecules to Aβ could shield the peptide loci relevant for membrane perturbation/penetration, preventing oligomer insertion. This hypothesis could be reasonable within the framework of the alternative mechanisms of oligomers/membrane interaction up to now proposed, where oligomers (i) bind to membranes inducing local perturbations; (ii) form pore structures, destabilizing cellular ionic homeostasis or (iii) bind to membrane receptors (Um et al., 2012; Jang et al., 2013; Sciacca et al., 2018; Pagano et al., 2019).

An unprecedented contribution to the comprehension of oligomer remodeling induced by natural compounds and of the molecular determinants of oligomer toxicity is offered by the research work of Melacini’s group, based on a combination of several cutting-edge NMR approaches, including ^15^N dark-state exchange saturation transfer, ^15^N transverse relaxation (^15^N R_2_), ^1^H based 2D saturation transfer difference (STDHSQC) experiments, with complementary techniques such as DLS, fluorescence, EM and wide-angle X-ray diffraction (WAXD) (Ahmed et al., 2017, 2019; Martinez et al., 2020).

The authors were able to show that EGCG remodels oligomers by weakening the monomer-protofibril contacts for the β1 strand peptide region (Q15–E22), which are critical for self-association, thus preventing further monomer addition and stabilizing smaller aggregates. At the same time, EGCG enhances monomer-protofibril interactions at the charged N-terminal region, which is important for the binding to lipid membranes.

**FIGURE 3:**
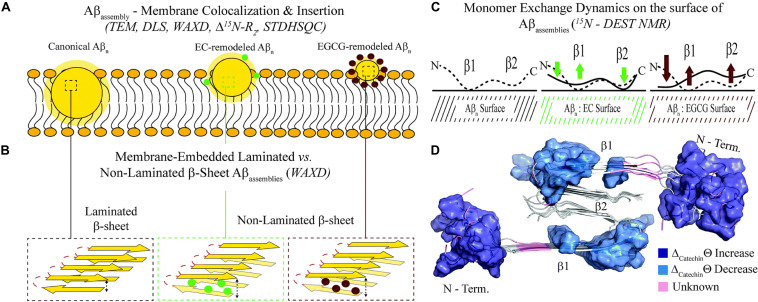
Proposed model for the molecular determinants of Aβ assembly toxicity. **(A)** Catechin-free oligomers (canonical Abn) insert and colocalize efficiently into the membrane due to their significant solvent exposure of hydrophobic surfaces (yellow glow surrounding Aβn). The catechin-remodeled oligomers, with less exposed hydrophobic sites, only insert into the membrane partially. **(B)** Both laminated and non-laminated cross-β-sheet structures can insert into the membrane, which indicates that cross-β-sheet structures are not required for membrane insertion. **(C)** The interaction of monomeric Aβ with toxic and remodeled oligomers is different within a membrane environment. EGCG-remodeled oligomers (maroon) show a significant disengagement of contacts with the b1 region and an opposite enhancement in the contacts with the N-terminal region, compared to untreated (black) oligomers. The EC-remodeled (green) oligomers exhibit a pattern at the N-terminus and b1 regions intermediate to the canonical and EGCG-remodeled oligomers, while a further enhancement in C-terminal contacts relative to both canonical and EGCG-remodeled Abn is observed. **(D)** In the Aβ40 fibril structure (PDB code: 2LMN) the residues that correlate with toxicity (blue) in the N-terminal and b1 regions can be found in the exterior of the fibril structure, while the b2 region not linked with toxicity is inaccessible to the environment (Ahmed et al., 2019). Published by The Royal Society of Chemistry.

The breakthrough of the research is the provision, for the first time, of a structure-toxicity relationship. The authors demonstrated that toxicity of the oligomers remodeled by different catechins scales proportionally to the enhanced solvent exposure of hydrophobic surfaces (Figure 3A). The combination of TEM, WAXD and ^15^N-based NMR experiments revealed key differences in the recognition of Aβ monomers within a membrane environment by the less toxic EGCG-remodeled Aβ and the more toxic canonical oligomer (Figures 3B–D). The integrated analyses of the data, through agglomerative clustering and Single Value Decomposition, allowed the authors to identify a cluster of molecular attributes unique to toxic oligomers, including surface hydrophobicity, oligomer size, shielding of the N-terminus and exposure of the β1 region to monomers. Interestingly, both N-terminus and β1-turn regions, which were identified as toxicity determinants of Aβ oligomers, are found in the Aβ fibril external regions, thus modulating the contacts and the subsequent insertion of Aβ oligomers into the membranes (Ahmed et al., 2019).

Interestingly, a good agreement is observed among the peptide loci proposed as toxicity determinants (Ahmed et al., 2019), with those deduced from the interaction studies of natural compounds with monomers and/or low molecular weight oligomers (Figure 2).

### Ability of Inhibitors to Cross Blood Brain Barrier and Strategies to Improve Their Bioavailability

In a therapeutical perspective, the most promising natural compounds are those that reach the brain tissue in a concentration sufficient to obtain beneficial effects. To this aim, two main factors must be taken in consideration: (i) the ability of the small molecule to cross the BBB; (ii) the compound bioavailability.

Most of the natural compounds discussed in this review have been characterized for their ability to cross the BBB (Table 2 and references therein). *In vitro* assays were employed for some molecules based on two model systems. The first is a BBB cellular kit, consisting of co-cultures of endothelial cells, pericytes, and astrocytes (Pervin et al., 2017). The second is a “parallel artificial membrane permeability assay” (PAMPA), which determines the permeability of substances from a donor compartment, through a membrane into an acceptor compartment (Simon et al., 2020). Some natural compounds were instead tested *in vivo*, administrating the compounds to mice through intraperitoneal or intra venous injection, and by oral administration. The subsequent analysis of brain tissues homogenates through chromatographic methods, allowed to quantify small molecule concentrations in the brain. In some instances, the efficiency of the compounds to cross BBB was only evaluated on the basis of behavioral tests in animals.

**TABLE 2:** Ability of natural compounds with antiaggregation effects on Aβ peptide to cross BBB.

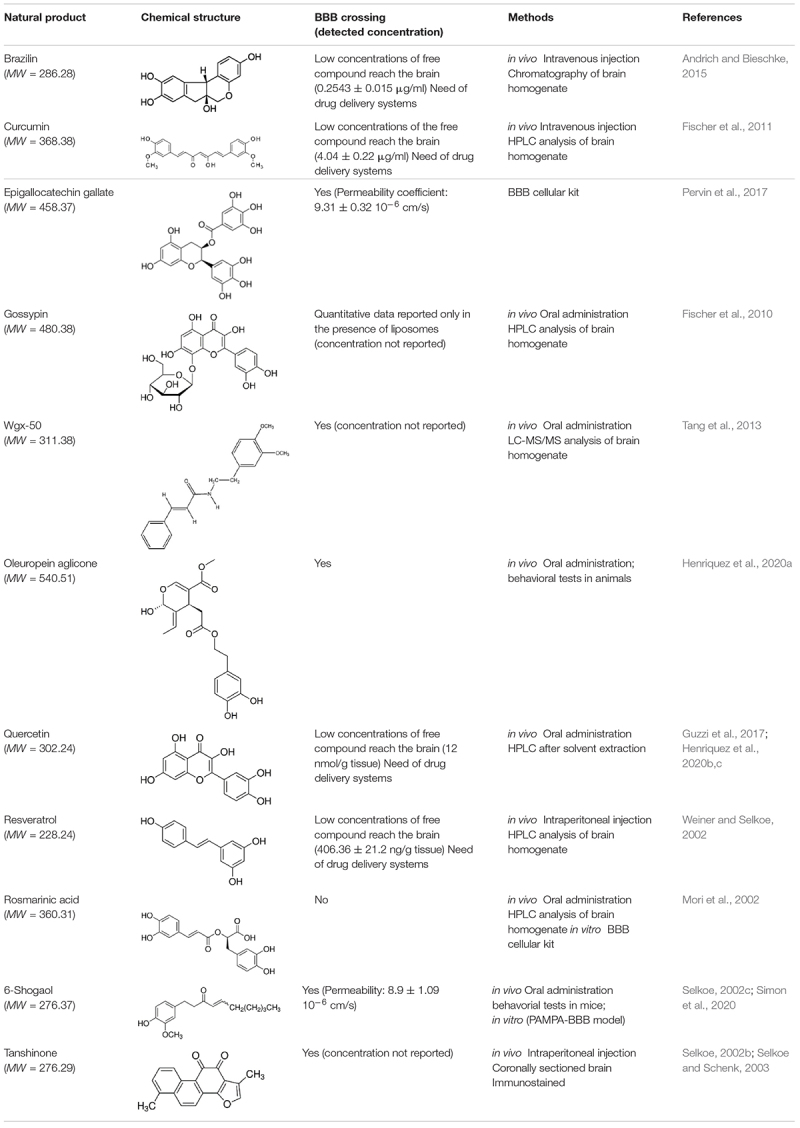

The majority of the molecules tested *in vivo* could cross the BBB, as expected on the basis of their low molecular weight (<500 Da) (Ramalho et al., 2020). The comparative analysis of the concentrations detected in the brain is difficult and is hampered by the different methods and/or units employed by the authors. However, most of the authors underlined that the concentrations reaching the brain were not sufficient to obtain beneficial therapeutic effects.

It is worth mentioning here that avoiding the BBB is the conception of a new potential strategy to treat AD, namely to design nanocarriers able to promote the so-called “sink-effect” (Matsuoka et al., 2003; Zhang and Lee, 2011; Tosi et al., 2019). The core of sink-effect hypothesis is based on the idea that brain and plasma Aβ pools are in equilibrium through the BBB and that the peripheral sequestration of Aβ may shift this equilibrium toward the peripheral blood circulation, eventually drawing out the excess from the brain and/or from the brain vessels. Results are reported supporting the hypothesis that Aβ oligomers spontaneously cross the BBB. Once reached the apical compartment, they are “stolen” by specifically tailored liposomes or polymeric NP decorated with anti-Aβ antibodies, thus shifting the equilibrium of the peptide from the brain to the peripheral circulation (Mancini et al., 2016; Carradori et al., 2018).

In any therapeutical approach, it is however clear that the main limiting factor is the low bioavailabity of the natural compounds, due to their chemical instability, rapid metabolism, and clearance from blood circulation (Wang et al., 1997; Craft et al., 2002; Shen and Ji, 2012; Liu et al., 2016).

A few strategies have been employed to overcome these limitations and develop neuroprotective drugs, efficiently inhibiting Aβ oligomerization. The first consists in embedding the natural compounds in carriers to enhance bioavailability and direct their distribution toward brain tissue (Ramalho et al., 2020). The investigated drug delivery systems include polymer-based and lipid-based nanoparticles, together with microemulsions and nanoemulsions. The choice of the best nanosystem depends on the physicochemical properties of the drug to be delivered and the drug delivery system properties, i.e., nanoparticle BBB crossing, clearance and induced toxicity (Saraiva et al., 2016; Patra et al., 2018). As an example, in the case of curcumin, which displays low water solubility, a rapid systemic elimination (Yang et al., 2007) and is scarcely capable to penetrate the BBB (Barbara et al., 2017), many drug delivery systems and formulations were investigated including liposomes, solid lipid microparticles, cyclodextrins, and nanoparticles using both natural or synthetic polymers (Barbara et al., 2017; Del Prado-Audelo et al., 2019). These formulations showed enhanced therapeutic properties compared to the free curcumin. Similar approaches were employed for quercetin and resveratrol (Ramalho et al., 2020). It is however, important to note that the prediction of the behavior of NPs *in vivo* still remains a subject of debate, and the “pros and cons” of a variety of NP are still being defined (Ordonez-Gutierrez and Wandosell, 2020; Ramalho et al., 2020).

An alternative strategy to enhance bioavailability is the synthesis of natural compounds derivatives. Chemical modifications have been successfully introduced in curcumin to remove labile moieties and improve the water solubility in physiological conditions, preserving the ability to bind Aβ oligomers and plaques (Airoldi et al., 2011). The chemical conjugation of the active natural compounds with a prodrug moiety that do not inactivate its inhibitory properties and effectively improve the molecule pharmacokinetic is a promising strategy applied for quercetin (Guzzi et al., 2017) and silybin (Garcia-Vinuales et al., 2020). In particular, glycoconjugation of silybin with trehalose resulted in a significant increase of the bioavailabity, with improved solubility and half-life in blood serum. NMR studies further demonstrated that the interaction of the glycoconjugated-silybin with Aβ oligomers is mainly mediated by silybin, indicating that the threalose moiety does not interfere with silybin interactions (Garcia-Vinuales et al., 2020). In this respect, NMR STD approaches provide important information to guide the effective choice of the prodrug and the linker for chemical conjugation.

Despite the attempted strategies, the bioavailability is still a major problem for the conversion of natural compounds into therapeutics and further innovative scientific and technological efforts are needed in this direction.

## Conclusion

Inhibiting the toxic aggregation of amyloidogenic Aβ peptides has been shown to be an attractive approach to fight against AD. Natural compounds that can act as modulators of amyloidogenic aggregation have been widely studied and a number of polyphenolic and non-polyphenolic inhibitors were shown to have powerful effects against protein aggregation through stabilizing monomers, inhibiting nucleation, disaggregating amyloid fibrils, and leading to off-pathway non-toxic oligomeric species.

The main goal of the research in the field, however, remains the identification of the molecular mechanism of natural compound action. An emerging concept that reconciles the large body of literature on the differing mechanisms of amyloid oligomer toxicity, is that toxicity is a pervasive property arising from the exposure of “toxic surfaces” shared by multiple soluble Aβ assemblies produced by the nucleation-dependent aggregation process (Selkoe, 2002a; Benilova et al., 2012).

Recent advancements in solution NMR, including methods to access the equilibria between NMR visible monomers and soluble high MW NMR invisible oligomers, such as DEST, combined to competitive ANS fluorescence and morphological DLS and EM data, allowed to propose the molecular determinants of Aβ assembly toxicity (Ahmed et al., 2019). Toxicity is regulated by the solvent exposure of hydrophobic surfaces. Changes in the accessibility of the hydrophobic β1-turn region and charged N-terminus to monomer/membrane recognition, have been shown to be the determinants of the induced toxicity (Ahmed et al., 2019). Most of the data reported in the literature converge to indicate these same regions as the preferred binding sites of natural compounds establishing interactions with NMR visible low molecular weight Aβ species. Interestingly N-term and β1 stretches are found in the external regions in both Aβ fibril structure and in the recently published tetramer structure (Ciudad et al., 2020). The structural data of the oligomeric species (Ciudad et al., 2020) together with those on structure-toxicity relationships (Ahmed et al., 2019) indicate, for the first time, that the critical features of the up to now elusive oligomeric species, responsible for AD pathology, are finally coming to light, thus making the design of effective AD inhibitors a realistic option.

Furthermore, some natural compounds, such as EGCG have been shown to interact with many amyloidogenic proteins, such as Aβ, αSyn, islet amyloid polypeptide (IAPP), huntingtin, tau, and immunoglobulin light chains, revealing that they act on aggregation through a similar mechanism (Andrich and Bieschke, 2015; Martinez et al., 2020). In addition to EGCG, also OleA and quercetin have been classified as potent polyphenols sharing common amyloid targets, namely Aβ, tau and α-Syn (Henriquez et al., 2020a). These observations strongly suggest that the structural knowledge of the mechanism of action of these natural compounds should be exploited as a starting point to design and develop therapeutic solutions for the prevention and treatment of different neurodegenerative diseases.

## Author Contributions

All authors contributed to conceiving the idea of this review, reviewed the literature, contributed to the writing and editing of this manuscript, and given approval to the final version of the manuscript.

## Conflict of Interest

The authors declare that the research was conducted in the absence of any commercial or financial relationships that could be construed as a potential conflict of interest.
